# Immature Neutrophil Phenotype and Enhanced Myeloperoxidase in Male Patients With Chronic Kidney Disease and Cardiovascular Comorbidity

**DOI:** 10.1111/apha.70290

**Published:** 2026-08-18

**Authors:** Sonja Vondenhoff, Alessandra Antwerpen, Carolina Victoria Cruz Junho, Philipp Martin, Sina Hourtz, Jinmi Zou, Berkan Kurt, Carolin Haas, Susanne Fleig, Corinna Schulte, Jane Lauxen, Martina Wessiepe, Ulrike Späte‐Schulze, Rafael Kramann, Jürgen Floege, Oliver Soehnlein, Joachim Jankowski, Claudia Goettsch, Yvonne Döring, Florian Kahles, Nikolaus Marx, Constance C. F. M. J. Baaten, Heidi Noels

**Affiliations:** ^1^ Institute for Molecular Cardiovascular Research (IMCAR), Uniklinik RWTH Aachen RWTH Aachen University Aachen Germany; ^2^ Department of Biochemistry, Cardiovascular Research Institute Maastricht Maastricht University Maastricht the Netherlands; ^3^ Department of Internal Medicine I Uniklinik RWTH Aachen Aachen Germany; ^4^ Institute of Physiology II University of Münster Münster Germany; ^5^ Department of Medicine II Uniklinik RWTH Aachen Aachen Germany; ^6^ Institute for Transfusion Medicine and Cell Therapeutics Uniklinik RWTH Aachen Aachen Germany; ^7^ Section of Geriodontics, Department of Conservative Dentistry and Periodontology, Center of Dental Medicine Jena University Hospital Jena Germany; ^8^ Institute of Experimental Pathology (ExPat), Center for Molecular Biology of Inflammation (ZMBE) University Hospital Münster, University of Münster Münster Germany; ^9^ Aachen‐Maastricht Institute for Cardiorenal Disease (AMICARE) Uniklinik RWTH Aachen Aachen Germany; ^10^ Department of Pathology, Cardiovascular Research Institute Maastricht Maastricht University Medical Centre Maastricht the Netherlands; ^11^ Institute of Physiology, Faculty of Medicine Carl Gustav Carus Technical University Dresden Dresden Germany; ^12^ Division of Angiology, Swiss Cardiovascular Center Inselspital, Bern University Hospital, University of Bern Bern Switzerland; ^13^ Department for BioMedical Research (DBMR) University of Bern Bern Switzerland; ^14^ DZHK (German Centre for Cardiovascular Research), Partner Site Munich Heart Alliance Munich Germany; ^15^ Institute for Cardiovascular Prevention (IPEK) Ludwig‐Maximilians‐University Munich (LMU) Munich Germany

**Keywords:** cardiorenal, chronic kidney disease, inflammation, maturation, neutrophils

## Abstract

**Aim:**

Patients with chronic kidney disease (CKD) have an increased cardiovascular risk. Since neutrophils and neutrophil‐borne proteins contribute to cardiovascular disease, we analyzed the neutrophil phenotype in patients with CKD who presented with a spectrum of cardiovascular comorbidities.

**Methods:**

Blood from two independent cohorts of male patients with moderate to advanced CKD recruited through the cardiology or nephrology unit was analyzed for neutrophils maturation and activation markers using flow cytometry compared to healthy controls. The formation of neutrophil‐extracellular traps was assessed using isolated neutrophils. Plasma levels of neutrophil‐borne proteins (neutrophil elastase, myeloperoxidase, and S100A8/A9), plasma inflammatory markers and cardiovascular disease characteristics were compared.

**Results:**

Both cohorts of CKD patients showed reduced neutrophil surface expression of maturation marker CD10 and higher plasma levels of myeloperoxidase compared to controls. No significant differences were observed in neutrophil surface activation markers in either baseline or stimulated conditions, or in the formation of neutrophil extracellular traps ex vivo. Although CKD patients presented with variable degrees of systemic inflammation and cardiovascular comorbidities, the immature neutrophil phenotype (low CD10) was not associated with either inflammatory status (CRP, IL6) or NT‐proBNP levels.

**Conclusion:**

Male cardiorenal patients with moderate to advanced CKD do not show altered neutrophil surface activation markers or ex vivo activation potential per se. Yet, they display a more immature neutrophil phenotype and higher circulating neutrophil‐borne myeloperoxidase as observed across a range of systemic inflammation degrees. This could potentially contribute to the overall increased cardiovascular risk in CKD.

## Practitioner Points

1. Patients with chronic kidney disease present with a high comorbidity burden of cardiovascular disease. Neutrophils play an important role in the pathophysiology of cardiovascular disease, including atherosclerosis, myocardial infarction, and heart failure.
2. Neutrophils from cardiorenal patients with moderate to advanced chronic kidney disease do not show differences in surface activation markers or ex vivo activation potential per se, but show a shift toward a more immature profile, paralleled by increased circulating levels of neutrophil‐borne myeloperoxidase.
3. This is observed in cardiorenal cohorts across a range of systemic inflammation and cardiovascular comorbidity burden and may contribute to the increased cardiovascular risk in these patients.

## 1 Introduction

Around 10%–15% of adults worldwide suffer from chronic kidney disease (CKD) and its prevalence is increasing due to an aging population and a rising incidence of CKD risk factors such as hypertension and diabetes [1, 2]. CKD is a progressive disease and is classified in five stages based on the remaining kidney function, expressed by the estimated glomerular filtration rate (eGFR) [3]. Whereas patients in CKD stage 1 and stage 2 display kidney damage with a normal (eGFR ≥ 90 mL/min/1.73 m^2^) or mildly reduced kidney filtration function (eGFR 60–89 mL/min/1.73 m^2^), patients in stage 3 (eGFR 30–59 mL/min/1.73 m^2^) and CKD stage 4 (eGFR 15–29 mL/min/1.73 m^2^) display a moderately to severely, or severely reduced kidney function, respectively. In CKD stage 5 (eGFR < 15 mL/min/1.73 m^2^), kidney function is drastically reduced, requiring dialysis (CKD5D) or kidney transplantation to survive [3].

Of note, CKD patients often display chronic low‐grade inflammation and have an increased cardiovascular risk, which manifests already in the early stages of CKD development [4, 5, 6]. As kidney function declines, cardiovascular risk further increases. While cardiovascular disease (CVD) presents in ~37.5% of persons with healthy kidney function, this is increased to 63.4%, 66.6%, and 75.3% of CKD patients in CKD stage 1–2, CKD stage 3 and CKD stage 4‐5D, respectively [6]. Around 40%–45% of patients suffering from advanced CKD die due to cardiovascular complications [7]. CVD manifests in CKD patients with an increased prevalence and progression of atherosclerosis as well as an increased risk of myocardial infarction and heart failure [8, 9, 10, 11, 12]. Of note, CVD can also contribute to CKD progression. This interplay between kidney and heart dysfunction is known as cardiorenal syndrome [13].

Neutrophils account for ~70% of circulating leukocytes and play an important role in the pathophysiology of CVD, including atherosclerosis and myocardial infarction [14]. Their production in the bone marrow and release into the circulation are tightly regulated, for example through the antagonistic action of the chemokine receptors CXCR2 and CXCR4. Low CXCR4 expression on neutrophils, a reduced concentration of the CXCR4 chemokine ligand CXCL12 in the bone marrow or increased CXCL12 plasma levels can trigger the release of neutrophils from the bone marrow into the circulation [15, 16, 17, 18, 19]. Other regulators of neutrophil production, survival and/or release from bone marrow include granulocyte colony‐stimulating factor (G‐CSF), granulocyte‐macrophage colony‐stimulating factor (GM‐CSF), serpin B1, the VLA‐4‐VCAM‐1 axis and chemokines as CXCL1 and CXCL2 [17, 18, 20, 21, 22, 23, 24].

Once neutrophils are released into the blood, they undergo natural phenotypical changes described as neutrophil aging. Based on the expression pattern of neutrophil cell surface receptors, neutrophils can be categorized according to their maturation and activation state [25]. In particular, the surface expression of CD10 is frequently used to distinguish between immature (CD10^negative^ neutrophils) and mature (CD10^positive^ neutrophils) neutrophils. Furthermore, CD11b, CD18, CD63 and CD66b are upregulated on the neutrophil surface upon neutrophil activation [26, 27, 28]. Neutrophils are well‐known for their pathogenic defense mechanisms including phagocytosis, the release of neutrophil extracellular traps (NETs) and degranulation. However, these mechanisms can also be activated in sterile, non‐infectious conditions and—when dysregulated or excessively activated—promote pathophysiological innate immune responses and disease, including CVD [14]. During neutrophil degranulation, different pro‐inflammatory proteins are released into the circulation, such as the neutrophil‐borne proteins neutrophil elastase and myeloperoxidase (MPO), which are both biomarkers of cardiovascular risk as well as contributors to cardiovascular pathophysiology [29, 30, 31, 32]. Both proteins are part of NETs, which exert pro‐inflammatory as well as prothrombotic effects [33, 34].

Overall, increased neutrophil counts and changes in neutrophil activation have been reported in CKD, but findings are conflicting and furthermore, most studies have focused on patients with advanced CKD and kidney failure (CKD stage 5D) [34, 35]. To provide insights into a potential neutrophil dysregulation in CKD without confounding effects of dialysis, this study analyzed the phenotype of peripheral blood neutrophils from hemodynamically stable, male CKD patients stage 3 and stage 4 (CKD3/4, 15 ≤ eGFR < 60 mL/min/1.73 m^2^) recruited through (1) the cardiology department and (2) the nephrology outpatient clinic in comparison to healthy controls. To examine the inherent impact of a (cardio)renal syndrome on neutrophils without the interference of an active infection or immunosuppressive therapy, patients were not eligible for study participation when they had an active infection, an active infectious or non‐infectious inflammatory disease (e.g., sarcoidosis or other granulomatous disease) or an auto‐immune disease, or if they were treated with systemic glucocorticoids or other immunosuppressive medication. Patients were also not eligible for study participation if they displayed a liver or intestinal disease, a hematopoietic malignancy, or an active or previous malignant disease within the past 5 years.

## 2 Results

### 2.1 Neutrophil Surface Activation Markers or NET Formation Capacity Are Not Altered but Male Cardiorenal Patients With Moderate to Advanced CKD Exhibit Lower Expression of the Maturation Marker CD10 and Higher Myeloperoxidase Plasma Levels

A first cohort of male patients with CKD stages 3–4 was recruited through the cardiology department, in parallel to a healthy male control cohort. Patients' characteristics, hematological parameters and medication are summarized in Tables 1 and 2. Typical comorbidities of the recruited patients included—in addition to CVD—hypertension and diabetes. Furthermore, the patients displayed a significantly reduced red blood cell count as well as a higher count of leukocytes and monocytes in peripheral blood compared to healthy male controls, consistent with previous reports [36, 37]. They also showed a trend toward an increased neutrophil count in blood (1.25‐fold increase; *p* = 0.14) (Table 1).

**TABLE 1:** Baseline clinical and hematological parameters of the cardiology‐recruited CKD cohort (Cohort 1) and respective healthy controls, as well as of the CVD cohort with eGFR ≥ 60 mL/min/1.73 m^2^.

	Healthy controls	CKD3/4 (Cohort 1) (# vs. controls)	CVD with eGFR ≥ 60 (# vs. controls; * vs. CKD)
*N*	10	21	12
Patient characteristics			
Age (years)	46 ± 13	76 ± 10 (0.0001 ####)	68 ± 12 (0.0022 ##)
Male (%)	100%	100%	100%
Creatinine (mg/dL)	n.d.	2.0 ± 0.6	1.0 ± 0.1 (0.0001 ****)
Urea (mg/dL)	n.d.	81 ± 41	38 ± 8 (0.0001 ****)
eGFR (mL/min/1.73 m^2^)	n.d.	35 ± 11	81 ± 14 (0.0001 ****)
WBC (×10^9^/L)	5.4 ± 1.3	6.9 ± 2.0 (0.0427 #)	7.3 ± 1.9 (0.0229 #)
RBC (×10^12^/L)	6.1 ± 1.1	4.5 ± 0.7 (0.0017 ##)	5.1 ± 0.4 (0.0361 #)
Neutrophils (×10^9^/L)	3.6 ± 1.1	4.5 ± 1.8 (0.14 n.s.)	4.6 ± 1.1 (0.0832 n.s.)
Eosinophils (×10^9^/L)	0.1 ± 0.1	0.2 ± 0.1 (0.6602 n.s.)	0.1 ± 0.1 (0.7096 n.s.)
Lymphocytes (×10^9^/L)	1.2 ± 0.4	1.5 ± 0.7 (0.3203 n.s.)	1.9 ± 0.8 (0.0475 #)
Monocytes (×10^9^/L)	0.5 ± 0.1	0.7 ± 0.1 (0.0023 ##)	0.7 ± 0.4 (0.1324 n.s)
Comorbidities			
Diabetes (%)	n.a.	48%	33%
Hypertension (%)	n.a.	90%	92%
CVD (%)	n.a.	100%	100%
Coronary artery disease		76%	67%
Heart failure		86%	58%
Time after acute CVD event (MI and stroke) (in months)	n.a.	91 ± 88	n.d.

*Note:* Hematological parameters were assessed in EDTA‐anticoagulated blood. Depicted are mean ± SD, and *p*‐values of comparison to healthy controls (#), using the Brown‐Forsythe and Welch ANOVA with Dunnett‘s T3 multiple comparison test for statistical analysis. For the CKD‐specific characteristics, the *p*‐values (*) indicate the comparison between the CVD and CKD cohort using the Welch's *t*‐test for statistical analysis. ^#^ *p* < 0.05; ^##^ *p* < 0.01; ^####/^****p < 0.0001.

Abbreviations: eGFR, estimated glomerular filtration rate; MI, myocardial infarction; n.a., not applicable; n.d., not determined; n.s., not significant; RBC, red blood cells; WBC, white blood cells.

**TABLE 2:** Likely CKD etiology and medication of the recruited CKD patient cohorts.

	CKD3/4 Cohort 1 (Cardiology‐recruited)	CKD3/4 Cohort 2 (Nephrology‐recruited)
*N*	21	6
Likely CKD etiology		
Glomerulonephritis plus systemic inflammatory disease	1 (4.8%)	0 (0%)
Diabetic nephropathy	6 (28.6%)	0 (0%)
Hypertension	9 (42.9%)	1 (17%)
Diabetic nephropathy + Hypertension	4 (19.0%)	1 (17%)
Other	1 (4.8%)	4 (66%)
Organ atrophy (shrunken kidney), likely due to renal artery stenosis or renal vein thrombosis with thrombophilia of unknown cause		2 (33%)
Nephrectomy due to malignancy		1 (17%)
Mixed cardiorenal/hepatorenal cause		1 (17%)
Medication		
Antihypertensive therapy		
ACE‐inhibitor	8 (38%)	1 (17%)
Thiazide diuretics	3 (14%)	2 (33%)
Beta blockers	15 (71%)	3 (50%)
ARNI	8 (38%)	2 (33%)
Lipid‐lowering therapy		
Statins	18 (86%)	4 (67%)
Ezetimibe	3 (14%)	4 (67%)
Erythropoietin	0 (0%)	0 (0%)
Vitamin D	4 (19%)	4 (67%)
Urea‐lowering therapy	8 (38%)	0 (0%)
Anticoagulants		
Phenprocoumon/Warfarin	3 (14%)	0 (0%)
DOAC	12 (57%)	3 (50%)
Antiplatelet therapy		
Acetylsalicylic acid	5 (24%)	3 (50%)
P2Y_12_ inhibitors	0 (0%)	0 (0%)
Diuretics		
MRA	3 (14%)	2 (33%)
Loop diuretics	15 (71%)	4 (67%)
Anti‐diabetic therapy		
Insulin	5 (24%)	0 (0%)
Metformin	2 (10%)	0 (0%)
Sulfonylureas	0 (0%)	0 (0%)
GLP‐1 receptor agonists	1 (5%)	0 (0%)
DPP‐4 inhibitors	2 (10%)	2 (33%)
SGLT2 inhibitors	13 (62%)	6 (100%)

*Note:* Indicated are number of patients and/or % of all patients or Mean ± SD. Likely CKD etiology was determined based on existing comorbidities and—where available—anatomical/structural kidney pathology.

Abbreviations: ACE, Angiotensin‐Converting Enzyme; CVD, cardiovascular disease; ARNI, Angiotensin Receptor–Neprilysin Inhibitor; DOAC, Direct Oral Anticoagulant; DPP4, Dipeptidyl Peptidase‐4; GLP‐1, Glucagon‐Like Peptide‐1; MRA, Mineralocorticoid Receptor Antagonist; n.a., not applicable; n.d., not determined; SGLT2, Sodium–Glucose Cotransporter 2.

**FIGURE 1:**
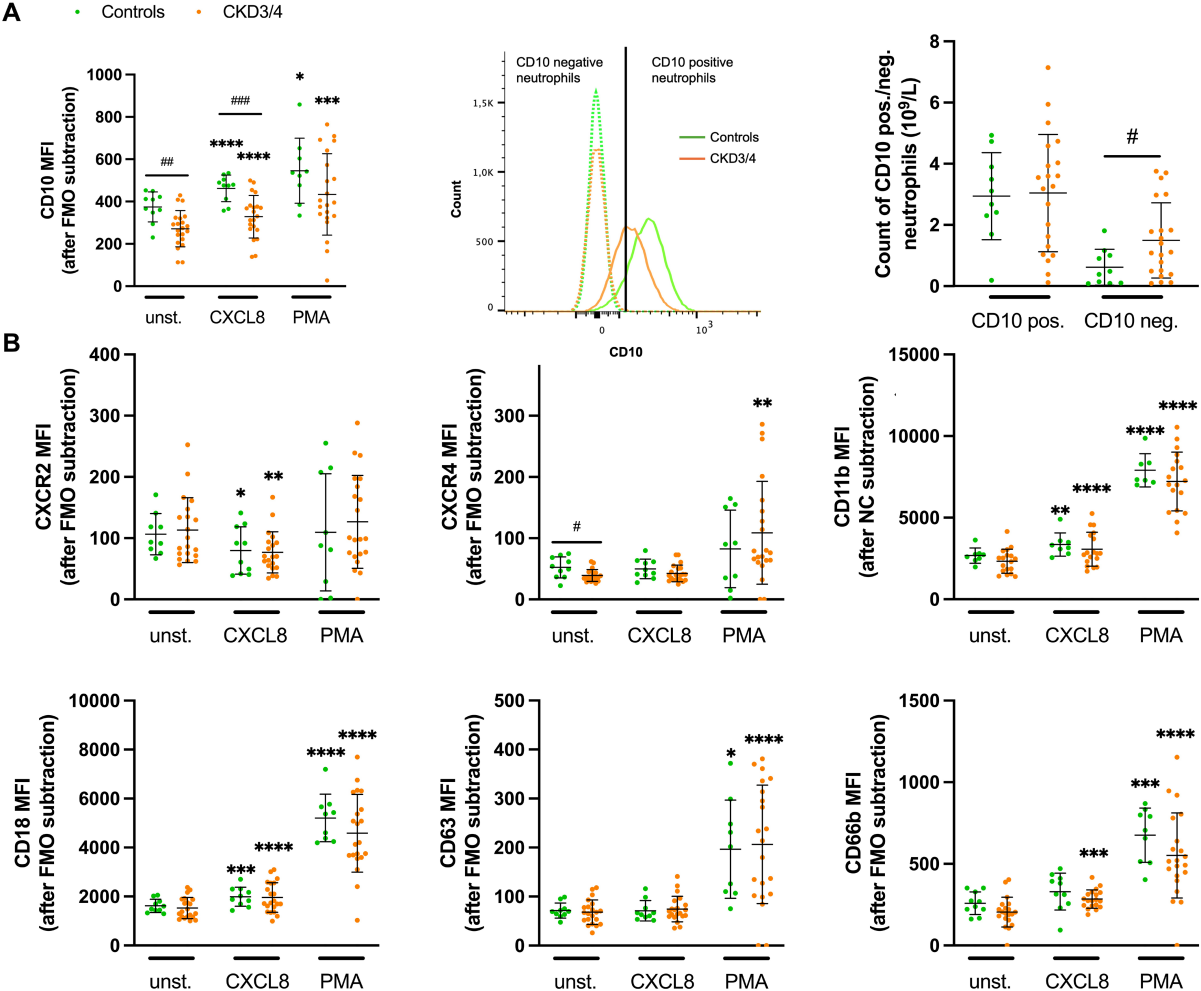
Emergence of immature neutrophil phenotype in patients with moderate to advanced CKD (Cohort 1). Male patients with CKD3/4 were recruited over the cardiology department (cohort 1). Peripheral whole blood neutrophils were analyzed in comparison to healthy male controls. (A, B) Quantification of markers on the surface of neutrophils in whole blood by flow cytometry, either in unstimulated conditions or upon stimulation with interleukin 8 (CXCL8, 50 ng/mL, 30 min) or phorbol 12‐myristate 13‐acetate (PMA, 50 ng/mL, 30 min). Displayed is mean ± SD of the geometric mean fluorescence intensity (MFI) after subtraction of the respective fluorescence minus one (FMO) control or of the respective fluorescence of the marker‐negative cell population (NC). (A) (Left) CD10 expression. (Middle) Representative histogram of CD10 expression and gating of neutrophils into CD10^positive^ and CD10^negative^ neutrophils (black line) based on the FMO control. The colored dotted lines represent the respective FMO control of healthy controls (green) and CKD3/4 (orange). (Right) CD10^positive^ versus CD10^negative^ neutrophil count in peripheral blood (unstimulated). (B) CXCR2, CXCR4 as well as neutrophil activation and maturation markers. (A, B) Parametric data; Mixed‐effect analysis with Tukey's multiple comparisons test, as appropriate. Comparison of stimulated to unstimulated cells within each patient/control group: Indicated by *; Comparison of patients to controls in either unstimulated or stimulated condition: Marked by #. *^/#^ *p* < 0.05; **^/##^ *p* < 0.01; ***^/###^ *p* < 0.001; ****p < 0.0001. (A, B) *n* = 7–10 for controls, *n* = 16–21 for CKD3/4. Differential n‐numbers among conditions or panels due to absence of PMA‐stimulation (for one donor), absence of FMO control for gating (for 3 donors for CD11b), or outlier exclusion based on ROUT outlier test (3 outlier exclusions for CXCR2 and CD66b).

Flow cytometric analysis of neutrophils in whole blood revealed under basal, unstimulated conditions a decreased surface expression of the neutrophil maturation marker CD10 in the patient cohort versus healthy donors (0.73‐fold, *p* = 0.0019), which persisted after stimulation with interleukin 8 (CXCL8, 50 ng/mL, 30 min) (Figure 1A, Figure S1). Additionally, quantification of CD10^positive^ and CD10^negative^ neutrophils revealed a significantly increased number of CD10^negative^ (immature) neutrophils in the CKD cohort compared to healthy donors. Within the CKD cohort, the number of CD10^negative^ neutrophils did not significantly correlate with the age of CKD patients (*r* = −0.33, *p =* 0.18). In parallel, surface expression of CXCR4—a retention marker that keeps neutrophils in the bone marrow [38]—showed a small but significant reduction in patients versus controls in basal conditions, although expression of its counterpart CXCR2 was not affected (Figure 1B). Stimulation of whole blood with phorbol 12‐myristate 13‐acetate (PMA, 50 ng/mL, 30 min) increased surface presentation of the activation markers CD11b, CD18, CD63 as well as CD66b on neutrophils of both groups. Stimulation with CXCL8 induced a small but significant increase in neutrophil CD11b and CD18 expression while reducing expression of its receptor CXCR2. However, no significant differences in expression levels were observed between both groups for any of these activation markers in either unstimulated or stimulated conditions (Figure 1B).

**FIGURE 2:**
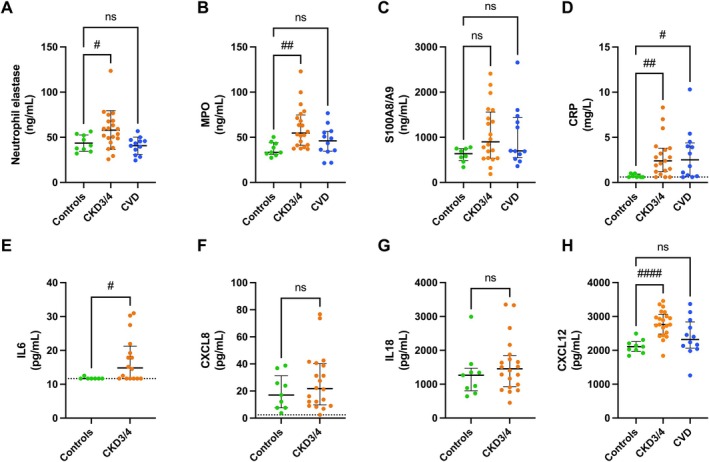
Analysis of circulating neutrophil‐borne proteins, chemotactic factors and inflammatory markers in CKD patients (Cohort 1) and CVD patients with eGFR ≥ 60 ml/min/1.73 m^2^. Male patients with CKD3/4 (cohort 1) as well as CVD patients with an eGFR ≥ 60 mL/min/1.73 m^2^ were recruited over the cardiology department. Plasma levels of neutrophil‐borne proteins, inflammatory proteins and CXCL12 were analyzed in comparison to healthy male controls. ELISA‐based quantification of: (A) neutrophil elastase, as mean ± SD, (B) myeloperoxidase (MPO), and (C) the S100A8/A9 complex, as median with interquartile range. (D) High‐sensitivity C‐reactive protein (CRP), as median with interquartile range. LegendPlex‐based quantification of: (E) interleukin 6 (IL6), (F) CXCL8 and (G) IL18, as median with interquartile range. (H) Chemokine CXCL12, as mean ± SD, via ELISA. The dotted horizontal lines indicate the detection limit. (A, H) Parametric data; Brown‐Forsythe and Welch ANOVA with Dunnett's T3 multiple comparison test. (B–G) Non‐parametric data; Mann–Whitney *U*‐test or Kruskal‐Wallis test with Dunn's multiple comparison test. ^#^ *p* < 0.05; ^##^ *p* < 0.01; ^####^ *p* < 0.0001; ns = not significant. (A–H) *n* = 7–9 for controls, *n* = 16–21 for CKD3/4, *n* = 12 for CVD. Differential *n*‐numbers among panels due to insufficient sample material or outlier exclusion based on ROUT test.

Nonetheless, neutrophil granule proteins in plasma were increased in the patients compared to controls. Myeloperoxidase (MPO) increased 1.64‐fold (*p* = 0.002), and neutrophil elastase 1.33‐fold (*p* = 0.033) (Figure 2A,B). The S100A8/A9 protein complex, which is also derived mainly from neutrophils [39] but from the cytoplasmic fraction, showed only a non‐significant increase (1.41‐fold; *p* = 0.187) (Figure 2C). Only neutrophil elastase showed a significant correlation with a more immature neutrophil signature based on the CD10^negative^ neutrophil count (*r* = 0.57, *p =* 0.003), also after adjustment for age (*p* = 0.006) (Table 3, Figures S2 and S3). To interrogate potential confounding effects of coexisting CVD or age, plasma samples from CVD patients of comparable age range as the CKD cohort but with an eGFR ≥ 60 mL/min/1.73 m^2^ were assessed simultaneously but no significant effects on neutrophil granule proteins were observed (Figure 2A–C, cohort characteristics in Table 1). Furthermore, a correlation analysis of age with neutrophil plasma markers independent of the influence of CKD revealed no association of advanced age with plasma levels of each of the neutrophil‐derived proteins in healthy controls and CVD patients (Figure S4). Also, in CKD patients, no positive correlation was detected between biological aging and neutrophil plasma markers or CD10^negative^ neutrophil count within the available age range (76 ± 10 years) (Figure S5). Finally, although diabetes affects neutrophil phenotype and accordingly leads to neutrophilia, increased formation of reactive oxygen species and NETs [40], our data did not show differences in neutrophil phenotypes between CKD patients without versus with diabetes (Figure S6).

**TABLE 3:** Correlation analysis of overall and CD10^negative^ neutrophil count with inflammatory markers, circulating neutrophil‐borne proteins and patient clinical data (Cohort 1).

	Neutrophil elastase	MPO	S100A8/A9	CRP	IL6	CXCL12	eGFR	Months after CVD event^a^	Eosinophils
CD10^negative^ neutrophil count (10^3^/μL)									
Unadjusted *r* (*p*)	0.57 (0.0031*)	0.26 (0.21)	0.08 (0.68)	0.08 (0.70)	0.02 (0.93)	0.30 (0.13)	−0.13 (0.61)	−0.04 (0.90)	0.14 (0.48)
Age‐adjusted *β* (*p*)	0.03 (0.0060*)	0.01 (0.29)	9.1* 10^−5^ (0.81)	−0.002 (0.98)	−0.02 (0.76)	0.0005 (0.35)	−0.02 (0.43)	0.002 (0.73)	2.4 (0.51)
Neutrophil count (10^3^/μL)									
Unadjusted *r* (*p*)	0.56 (0.0016*)	0.29 (0.12)	0.56 (0.0018*)	0.26 (0.19)	0.23 (0.30)	−0.13 (0.51)	0.20 (0.40)	0.02 (0.95)	−0.07 (0.73)
Age‐adjusted *β* (*p*)	0.048 (0.0019*)	0.02 (0.13)	0.0016 (0.0022*)	0.22 (0.20)	0.069 (0.29)	−0.0008 (0.33)	0.032 (0.36)	0.0057 (0.40)	−1.86 (0.73)

*Note:* Included were both controls and CKD patients, except for eGFR and months after CVD (patients only). Row 1: Analysis using unadjusted simple linear regression: Two‐tailed Pearson correlation coefficients test, with correlation coefficient *r* and *p*‐value. Row 2: Analysis using multiple linear regression with adjustment for age, with regression coefficient *β* and *p*‐value. Associated graphs in Figures S2 and S3.

Abbreviations: CRP, high‐sensitivity C‐reactive protein; eGFR, estimated glomerular filtration rate; IL6, interleukin 6; MPO, myeloperoxidase; S100A8/A9, S100 calcium‐binding protein A8/A9 protein complex.

^a^ Months after acute CVD event (non‐fatal myocardial infarction or stroke).

* *p* < 0.01.

Since MPO, neutrophil elastase and S100A8/A9 are also components of neutrophil‐derived extracellular traps (NETs) and in part secreted during NET formation, we analyzed the DNA release capacity of isolated neutrophils after 3 h. Neutrophils responded with increased DNA release to PMA (but not CXCL8); however, there were no significant differences between the patients and controls in either unstimulated or stimulated conditions (Figure S7A–C). Of note, isolated neutrophils from CKD patients did not show any signs of cell death as revealed by Sytox staining, thus excluding increased plasma levels of neutrophil proteins due to issues with sample preparation (Figure S7D).

**FIGURE 3:**
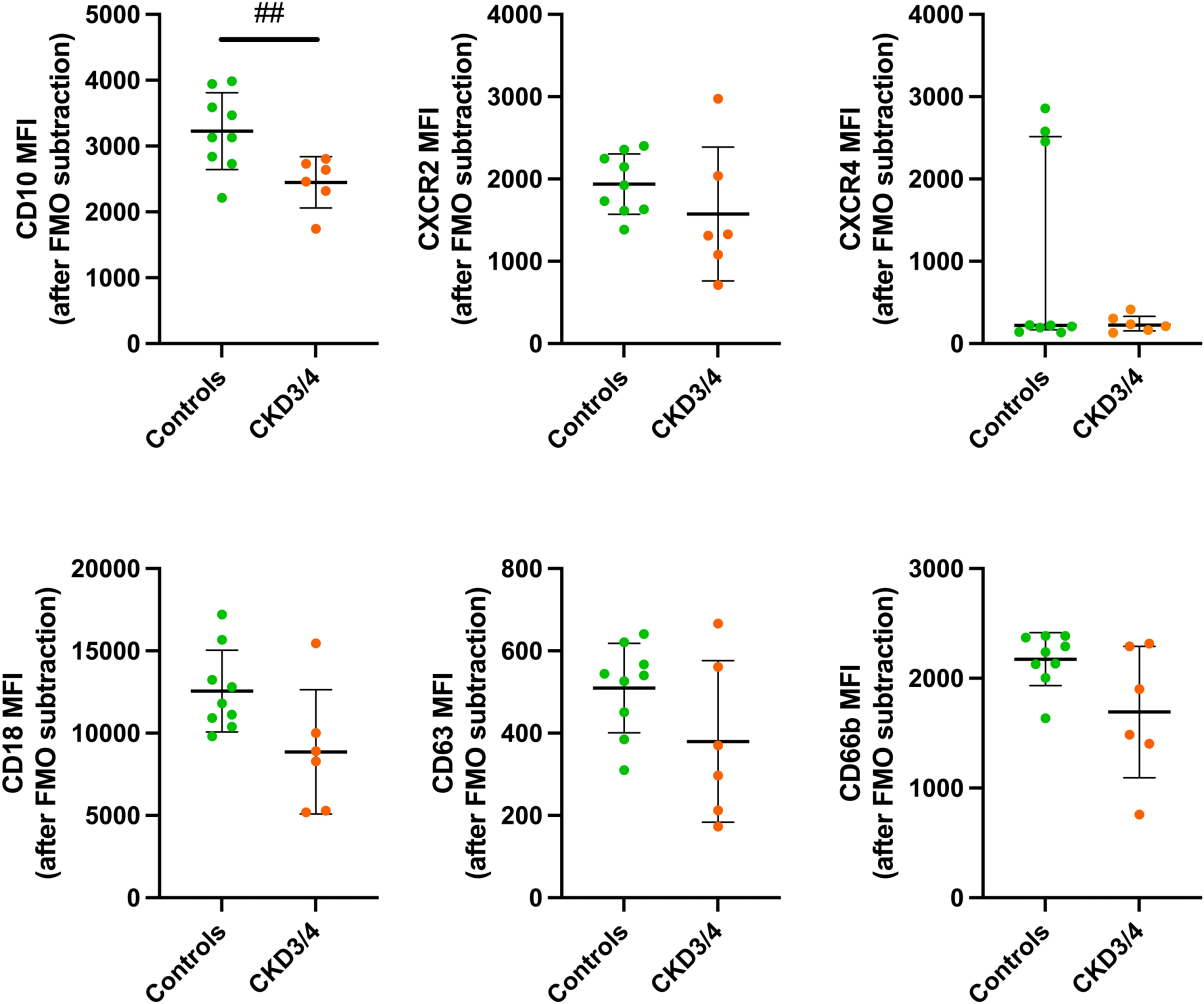
Emergence of immature neutrophil phenotype in patients with moderate to advanced CKD (Cohort 2). Male patients with CKD3/4 were recruited over the nephrology department (cohort 2). Peripheral whole blood neutrophils were analyzed in comparison to age − matched male controls presenting to the blood bank for routine blood donation and meeting standard blood‐donor eligibility criteria (Table 3). Quantification of markers on the surface of neutrophils in whole blood by flow cytometry in unstimulated conditions. Displayed is mean ± SD or median with interquartile range (CXCR4) of the geometric mean fluorescence intensity (MFI) after subtraction of the respective fluorescence minus one (FMO) control. CD10, CXCR2 and CXCR4 as well as neutrophil activation and maturation markers are shown. CXCR4: Non‐parametric data; Mann–Whitney test. Others: Parametric data; Welch's *t*‐test, as appropriate. ^##^ *p* < 0.01. *n* = 9 for controls, *n* = 6 for CKD3/4.

**FIGURE 4:**
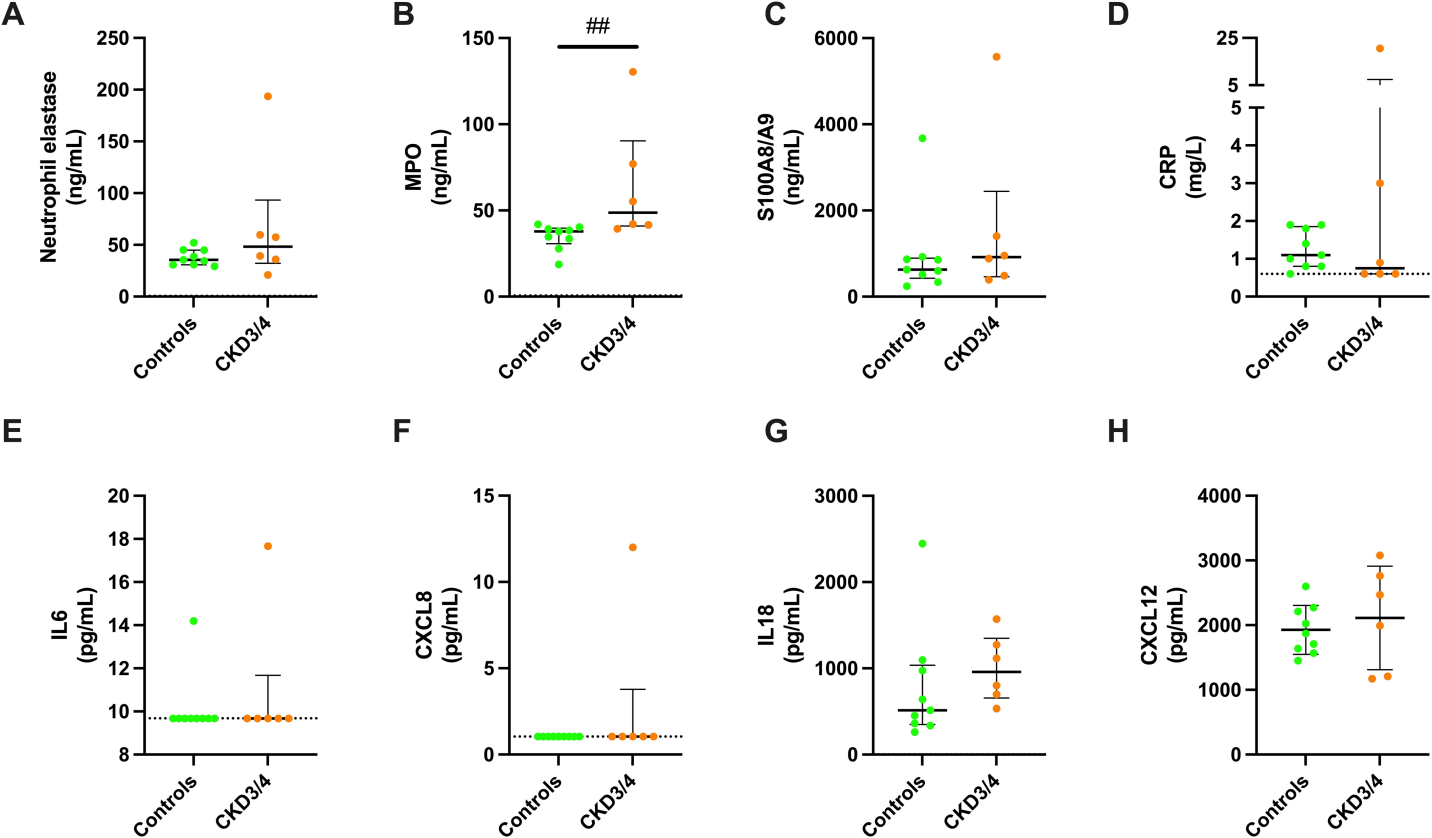
Analysis of circulating neutrophil‐borne proteins, chemotactic factors and inflammatory markers in CKD patients (Cohort 2). Male patients with CKD3/4 were recruited over the nephrology department (cohort 2). Plasma levels of neutrophil‐borne proteins, inflammatory proteins and CXCL12 were analyzed in comparison to age‐matched male controls presenting to the blood bank for routine blood donation. ELISA‐based quantification of: (A) neutrophil elastase, (B) myeloperoxidase (MPO), and (C) the S100A8/A9 complex, as median with interquartile range. (D) High‐sensitivity C‐reactive protein (CRP), as median with interquartile range. LegendPlex‐based quantification of: (E) interleukin 6 (IL6), (F) CXCL8 and (G) IL18, as median with interquartile range. (H) Chemokine CXCL12, as mean ± SD, via ELISA. The dotted horizontal lines indicate the detection limit. (A–G) Non‐parametric data; Mann–Whitney test. (H) Parametric data; Welch's *t*‐test. ^##^ *p* < 0.01; *n* = 9 for controls, *n* = 6 for CKD3/4.

To validate our findings, a second independent cohort of male patients with CKD3‐4 was recruited through the nephrology department. In parallel, an age‐matched male control cohort was recruited among volunteers at the Aachen blood bank. Patient characteristics were mostly comparable to those in the first cohort (Tables 2 and 4), except that no significant differences in red blood cell and monocyte count were detected, mainly due to differences in baseline levels among the controls. Also, both leukocytes and neutrophils were significantly increased in the CKD patients (leukocytes: 1.36‐fold increase; *p* = 0.02; neutrophils: 1.47‐fold increase; *p* = 0.03). Analysis of this second cohort supported the significantly lower surface expression of CD10 on neutrophils of CKD patients compared to controls (0.76‐fold, *p* = 0.0085) (Figure 3). No significant differences were observed for any of the other cell surface proteins comparing neutrophils of both groups in basal conditions (Figure 3). Only MPO was significantly elevated in blood of the nephrology‐recruited CKD patients (1.29‐fold; *p* = 0.0028), without differences in neutrophil elastase or S100A8/A9 (Figure 4).

**TABLE 4:** Baseline clinical and hematological parameters of the nephrology‐recruited cohort (Cohort 2) and respective healthy controls.

	Controls	CKD3/4 Cohort 2
*N*	9	6
Patient characteristics		
Age (years)	65 ± 7	62 ± 12 (0.64 n.s.)
Male (%)	100%	100%
Creatinine (mg/dL)	n.d.	2.2 ± 0.4
Urea (mg/dL)	n.d.	75.6 ± 27.0
eGFR (mL/min/1.73 m^2^)	n.d.	33.0 ± 8.5
WBC (×10^9^/L)	6.4 ± 1.5	8.7 ± 1.6 (0.02 #)
RBC (×10^12^/L)	4.9 ± 0.7	5.1 ± 1.2 (0.75 n.s.)
Neutrophils (×10^9^/L)	4.1 ± 1.2	6.1 ± 1.5 (0.03 #)
Eosinophils (×10^9^/L)	0.3 ± 0.2	0.3 ± 0.2 (0.92 n.s.)
Lymphocytes (×10^9^/L)	1.4 ± 0.3	1.6 ± 0.4 (0.22 n.s.)
Monocytes (×10^9^/L)	0.6 ± 0.1	0.7 ± 0.2 (0.20 n.s.)
Comorbidities		
Diabetes (%)	n.d.	33%
Hypertension (%)	n.d.	100%
CVD (%)	n.d.	66%
Coronary heart disease		33%
Heart failure		33%

*Note:* The control cohort included sex‐ and age‐matched volunteers who presented to the local blood bank in the city center for routine blood donation and met standard blood‐donor eligibility criteria. Hematological parameters were assessed in EDTA‐anticoagulated blood. Depicted are mean ± SD, and *p*‐values of comparison to the control group (Welch's *t*‐test).

Abbreviations: eGFR, estimated glomerular filtration rate; n.d., not determined; n.s., not significant; RBC, red blood cells; WBC, white blood cells.

^#^ *p* < 0.05.

Taken together, patients with moderate to advanced CKD consistently displayed a lower expression of the neutrophil maturation marker CD10 and increased circulating MPO, without differences in neutrophil surface activation markers, ex vivo activation potential or NET formation capacity.

### 2.2 Inflammatory Profiles and Cardiovascular Comorbidity Burden in CKD Patients Do Not Correlate With the Immature Neutrophil Counts

To identify a potential link between neutrophil maturation level (CD10) and the patients' inflammatory profile, we next analyzed parameters of systemic inflammation. The CKD patients recruited over the cardiology department (cohort 1) displayed a state of enhanced systemic inflammation, with increased plasma levels of C‐reactive protein (CRP) and IL6—but not CXCL8 or IL18—compared to controls (Figure 2D–G). The age‐matched CVD cohort showed comparably high and significantly increased CRP plasma levels (Figure 2D; Median and interquartile range for CKD: 2.4 mg/L (1.2–3.8) vs. CVD: 2.5 mg/L (0.73–4.38); *p* > 0.99). Plasma levels of CXCL12 were significantly increased in the CKD cohort, but not in the CVD cohort (Figure 2H). Despite the increased inflammatory status of the cardiology‐recruited CKD cohort, no association was observed between either the inflammation markers (CRP, IL6) or the CXCL12 plasma levels and the count of either CD10^negative^ neutrophils (for CXCL12: *r* = 0.30, *p =* 0.13) or all neutrophils (for CXCL12: *r* = −0,13, *p =* 0.51) (Table 3, Figures S2B and S3B). Plasma levels of granulocyte colony‐stimulating factor (G‐CSF) as important regulator of neutrophil mobilization from the bone marrow to the periphery [19], especially under stress conditions, were below the detection limit in all samples (data not shown).

The CKD cohort recruited through the nephrology division (cohort 2) did not show any significant difference in plasma levels of inflammatory markers (CRP, IL6, CXCL8 or IL18) or CXCL12 compared to the control group (Figure 4D–H), indicating a lower inflammatory status than observed in the cardiology‐recruited CKD cohort.

**FIGURE 5:**
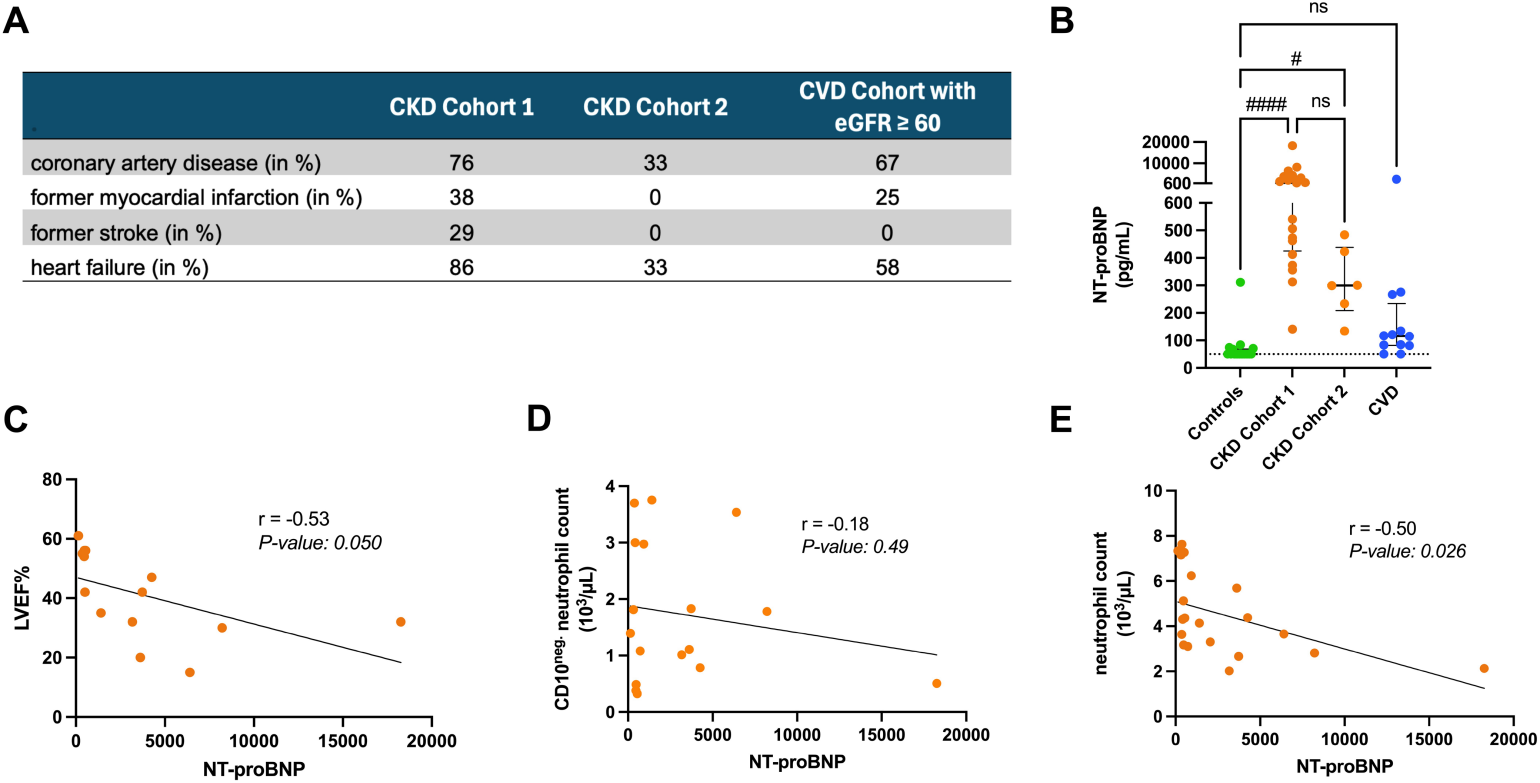
Comparison of parameters of cardiovascular disease status in the recruited patient cohorts. Male patients with CKD3/4 from the cardiology department (cohort 1) or the nephrology department (cohort 2) and male CVD patients with an eGFR≥ 60 mL/min/1.73 m^2^ were assessed regarding their cardiovascular burden, in comparison to healthy male controls. (A) Overview table of persistent CVD diseases in the respective cohorts, displayed as percentage. (B) NT‐pro‐BNP in plasma as marker of myocardial stress displayed as median + interquartile range. Detection limit indicated as horizontal dotted line. Non‐parametric data: Kruskal Wallis test with Dunn's multiple comparisons test. ^#^ *p* < 0.05; ^####^ *p* < 0.0001; ns = not significant; *n* = 18 for Controls, *n* = 20 for CKD Cohort 1, *n* = 6 for CKD Cohort 2, *n* = 12 for CVD cohort. One sample from Cohort 1 and CVD cohort was absent due to insufficient sample material. (C–E) Correlation analysis of NT‐proBNP levels of Cohort 1, with (C) left ventricular ejection fraction (LVEF) in %, (D) CD10^negative^ neutrophil count and (E) total neutrophil count in peripheral blood. *n* = 14–20; statistical analysis with two‐tailed Spearman correlation coefficients test.

To explore potential underlying differences in clinical characteristics and considering the increased risk of both atherosclerotic and non‐atherosclerotic CVD, including heart failure, in CKD [4, 7, 8], the cardiovascular status of both CKD cohorts was compared. The cardiology‐recruited cohort (cohort 1) contained hemodynamically stable patients with CKD and CVD, who either visited the outpatient clinic (29%) or were hospitalized for diagnostic and/or therapeutic interventions for their underlying cardiovascular condition (71%, full details in methods description). The nephrology‐recruited CKD cohort (cohort 2) was recruited from the nephrology outpatient clinic and showed a CVD comorbidity status in 4 of 6 patients (66%). The cardiology‐recruited cohort had a higher prevalence of cardiovascular comorbidities (including atherosclerosis‐related CVD and heart failure) compared to the nephrology‐recruited CKD cohort (Figure 5A). Furthermore, whereas the CVD cohort with an eGFR ≥ 60 mL/min/1.73 m^2^ displayed a small but non‐significant increase in circulating levels of NT‐proBNP as marker of myocardial stress, both CKD cohorts presented significantly elevated NT‐proBNP levels compared to the controls, with the strongest increase observed for the cardiology‐recruited CKD cohort (Figure 5B). NT‐proBNP levels were inversely correlated with left ventricular ejection fraction and—unexpectedly—with total neutrophil count, but not with the count of CD10^negative^ neutrophils in peripheral blood (Figure 5C–E).

In summary, the recruited CKD patients presented with a variable degree of systemic inflammation and cardiovascular comorbidities, but a direct association of CRP, IL6 or NT‐proBNP with the immature (low CD10) phenotype of neutrophils could not be detected.

## 3 Discussion

CVD is a highly prevalent condition in CKD patients [8]. Our study reveals that male cardiorenal patients with moderate to advanced CKD do not show altered neutrophil surface activation markers, ex vivo activation potential or NET formation per se. Yet, they display a more immature neutrophil phenotype along with increased circulating neutrophil‐borne myeloperoxidase, as observed across a range of systemic inflammation levels.

In a first cohort of male cardiorenal patients with moderate to advanced CKD (15 ≤ eGFR < 60 mL/min/1.73 m^2^) recruited through the cardiology department, we observed a trend toward an increased number of circulating neutrophils and a reduced state of neutrophil maturation, illustrated by an increased number of CD10^negative^ neutrophils in blood and a reduced neutrophil CD10 surface expression. Of note, CD10^negative^ neutrophils are also markedly increased in patients with unstable angina and ST‐segment elevation myocardial infarction [41]. However, although the CKD patients included in this cohort all also suffered from CVD, both patients who visited the outpatient clinic or were hospitalized at the cardiology department were hemodynamically stable and had no acute cardiovascular event (non‐fatal myocardial infarction or stroke) within at least 5 months before blood collection. Also, the hospitalized patients enrolled in this study donated blood either before any major medical examinations (in case of e.g., hospitalization for the evaluation and management of aortic valve disease or cardiac arrhythmias), or after successful treatment and clinical stabilization in case of initial hospitalization for cardiac decompensation. Also, a correlation analysis could not detect an association between CD10^negative^ neutrophil count and months after a prior acute CVD event (non‐fatal myocardial infarction or stroke), nor with NT‐proBNP as marker of myocardial stress. In addition, the lower CD10 surface expression of blood neutrophils in CKD was supported in a second independent cohort of CKD patients recruited through the nephrology department, who—in comparison with the cardiology‐recruited cohort—presented with a lower cardiovascular burden as demonstrated by the clinical characterization of cardiovascular comorbidities as well as NT‐proBNP levels in blood. This patient cohort also did not display a strong systemic pro‐inflammatory profile based on CRP and IL6 levels, different from the cardiology‐recruited cohort. Combined, this supports a contribution of CKD or a cardiorenal status rather than solely (acute) CVD or systemic inflammation to the enhanced immature phenotype of neutrophils observed in blood of both CKD cohorts.

Regarding CD10 surface expression, we showed that stimulation with either CXCL8 or PMA increased the surface expression of CD10 on both healthy and CKD neutrophils. Whereas one older study reported a downregulation of CD10 upon high PMA stimulation (6 times higher PMA concentrations as used in our study) [42], other studies similarly identified an upregulation of CD10 upon stimulation of neutrophils with lipopolysaccharide [43, 44] or complement factor C5 [45]. Recently, a CD11b^high^ CD10^high^ neutrophil subpopulation with an increased activation status was identified in portal blood of patients with alcoholic liver disease and portal hypertension, with CD10 surface expression shown to be triggered by strong endotoxin stimulation [44]. In contrast to these highly inflammatory patients, our study—which excluded patients with an acute high inflammation or active infection—consistently detected reduced neutrophil CD10 expression in CKD cohorts irrespective of systemic inflammatory status, and this along with increased plasma levels of the pro‐inflammatory neutrophil granule protein MPO. These findings suggest that in CKD, the reduced neutrophil CD10 expression is unlikely to reflect an overall reduced neutrophil activation state. Since MPO is relatively large (being multimeric with two 55 kDa and two 15 kDa subunits), impaired kidney clearance is unlikely to account for the increased plasma MPO levels observed in CKD, and instead a previous study suggested a key role for macrophages in MPO clearance [46]. Since we did not find any of the assessed neutrophil surface activation markers to be altered in CKD under basal or stimulated conditions, higher plasma MPO levels may instead result from transient neutrophil activation, local inflammatory niches (e.g., inflamed vasculature, with endothelial dysfunction increased in CKD [8]) or release from a small, activated neutrophil subpopulation, rather than from a global systemic neutrophil activation per se. In this context, the shift to CD10^low^ neutrophils, together with elevated circulating neutrophil counts, more likely reflects enhanced mobilization of immature neutrophils from the bone marrow into the peripheral circulation. The absence of a correlation between CXCL12 levels and the CD10^negative^ neutrophil number in blood and the even greater increase in peripheral neutrophils in the nephrology CKD cohort without concomitant changes in the CXCL12/CXC4 axis suggest that additional, yet unknown mechanisms are likely involved. This could include a potential modulation of the bone marrow environment itself, as shown for other chronic inflammatory diseases [47].

Overall, the neutrophil‐derived proteins MPO, neutrophil elastase and S100A8/A9 are implicated in the pathophysiology of CVD including vascular inflammation and atherosclerosis [8, 14]. For example, free MPO in the blood is thought to be responsible for vascular damage by generating harmful oxidants, triggering endothelial dysfunction and attracting neutrophils to the vessel wall [30, 48]. Also, released neutrophil elastase can enhance vascular permeability and mediate inflammatory cell adhesion [32, 49]. Beyond pro‐inflammatory effects of neutrophils and neutrophil‐borne proteins, an immature neutrophil phenotype has been associated with vascular calcification in CKD5 patients undergoing dialysis [50], which also increases cardiovascular risk in CKD patients [51]. Thus, the altered neutrophil phenotype that we identified in patients with moderate to advanced CKD may further contribute to their increased cardiovascular risk. Of note, as recently discussed, the overall higher NT‐proBNP levels detected in both CKD cohorts, exceeding even those seen in the CVD cohort with an eGFR ≥ 60 mL/min/1.73 m^2^, can be partly, but not exclusively, attributed to reduced kidney clearance, with increased secretion upon cardiomyocyte stress being a likely additional contributing factor. The same NT‐proBNP concentration even predicted a higher absolute risk of adverse outcomes for people with heart failure and reduced kidney function, compared with heart failure patients with preserved kidney function [52].

Study limitations are the small and all male cohorts of CKD patients, with patient eligibility strongly limited due to strong exclusion criteria of for example an active inflammation, auto‐immune disease or anti‐inflammatory medication. The ultimate focus on males in this study was based on highest male patient availability and may partly be due to the initial recruitment over the cardiology department, with men having an earlier CVD onset and higher overall CVD hospitalization rate [53]. Furthermore, both recruited CKD cohorts had pronounced cardiovascular comorbidity. While difficult to avoid in the stage of moderate‐to‐advanced CKD given the strong kidney‐heart pathophysiology crosslink [4, 5, 6, 12], initial recruitment through the cardiology unit inherently biased selection toward CKD patients with prominent cardiorenal disease. Yet, for the observed effects on plasma levels of neutrophil‐derived proteins, the additional integration of a second, independent CKD cohort recruited through the nephrology unit and with a lower cardiovascular comorbidity burden, as well as the additional comparison with a CVD cohort without kidney dysfunction, enabled us to distinguish between mainly CKD/cardiorenal versus purely CVD‐driven effects. Instead, a CVD contribution to the observed effects on CD10 cannot be excluded, since an analysis of neutrophil CD10 expression in the CVD cohort without kidney dysfunction was not possible due to availability of only plasma samples for this study. Furthermore, although the inclusion of an age‐matched CVD cohort and a second small CKD cohort with an age‐matched control group collectively provide first support that age did not confound our findings, including an age‐matched healthy control group also in the first CKD cohort would have been required to definitively rule out an impact of age. Differential medication use (e.g., antihypertensive therapy, antithrombotic therapy), hypertension or smoking may have contributed to data variability. Finally, the measurement of eGFR in our study is based solely on creatinine; the additional use of cystatin C as well as albumin/creatinine ratio in urine could have further improved CKD stage classification [54].

## 4 Conclusion

In conclusion, this study reveals a more immature (low CD10) neutrophil phenotype along with increased levels of the pro‐inflammatory neutrophil‐borne MPO in blood of male cardiorenal patients with moderate to advanced CKD. This was consistently observed in cohorts with different ranges of systemic CRP and IL6 levels and may contribute to and accelerate the development and progression of CVD. In addition, this study highlights the variable degree of systemic inflammation (CRP, IL6) and the high CVD comorbidity burden in CKD patient cohorts, with potential impact on study readouts. This underlines the importance of categorizing both CKD, CVD and systemic inflammatory status in studies examining the immune system. Future studies should address in larger patient cohorts the potential impact of CKD on leukopoiesis, and specifically also neutrophil generation and maturation, in bone marrow and expand analysis to female participants.

## 5 Materials and Methods

### 5.1 Study Design and CKD Patient Population

The study was approved by the local Medical Ethics Committee (EK418/21, EK473/21, University Hospital RWTH Aachen). Written informed consent according to the Declaration of Helsinki was obtained from all participants. Upon consent, CKD patients were recruited at the cardiology or nephrology departments of the University Hospital RWTH Aachen. Patients who were ≥ 18 years of age, hemodynamically stable and diagnosed with CKD stage 3 or 4 were eligible for study participation. Patients were excluded from study participation if treated with glucocorticoids or other immunosuppressive medication, if displaying an active infection, an active infectious or non‐infectious inflammatory disease (e.g., sarcoidosis or other granulomatous disease), an auto‐immune disease, a liver or intestinal disease, a hematopoietic malignancy, or an active or previous malignant disease within the past 5 years.

#### 5.1.1 Sample Collection Through the Cardiology Department (Cohort 1)

Upon consent, eligible CKD patients who visited the outpatient clinic (29%) of the cardiology department of the University Hospital RWTH Aachen (Medical Clinic 1) or who were hospitalized (71%) at the cardiology department, were included in the period from December 2023 until June 2024. The hospitalized patients included hemodynamically stable patients who received diagnostic and/or therapeutic interventions for their underlying cardiovascular condition during their hospital stay, for example evaluation and management of aortic valve disease (47%), cardiac arrhythmias (33%) or coronary angiography or exclusion of acute coronary syndrome (13%). These patients were enrolled in the study immediately upon admission, and blood samples were taken before any major medical examinations. One patient was included following treatment for cardiac decompensation (7%). Patient enrollment was restricted to a period of clinical stability and did not occur during the initial acute management phase to minimize confounding effects of acute illness on the study outcomes.

Blood samples from sex‐matched healthy volunteers were collected via the local blood bank in the hospital; these donors were anonymized upon study inclusion with only age and sex recorded. Of note, healthy donors participating in blood donation must complete a standardized questionnaire to rule out any relevant pre‐existing conditions. This prevents study inclusion in case of active or chronic diseases (severe heart or blood vessel diseases, serious disorders of the central nervous system, clinically relevant blood clotting disorders, repeated fainting or seizures, other serious active or chronic diseases affecting the digestive, urinary, blood, immune, metabolic, kidney, or respiratory systems), current or past malignant cancers, diabetes treated with insulin, chronic or transmissible infections (e.g., HIV, hepatitis B/C…), an active infection, or when being within four weeks after a feverish illness or within one week after an uncomplicated infection.

In the morning, K_3_‐EDTA, 3.2% (w/v) citrate and lithium‐heparin anticoagulated blood was collected via venipuncture using a 21G needle (Sarstedt, Nümbrecht, Germany) into S‐monovette tubes (Sarstedt, Nümbrecht, Germany). After the blood sample was taken, notification was given immediately so that the blood could be collected and processed without delay (approx. 10 min from collecting to processing the blood). A complete whole blood cell count was measured in EDTA‐anticoagulated blood using a Sysmex XN350 analyzer (Kobe, Japan). Plasma was prepared from EDTA‐anticoagulated blood through one‐time centrifugation at 2150 g for 10 min and stored directly at −80°C for further use. Citrate anticoagulated blood was used for neutrophil analysis in whole blood. Lithium‐heparin anticoagulated blood was used for neutrophil isolation.

#### 5.1.2 Sample Collection Through the Nephrology Department (Cohort 2)

Upon consent, eligible CKD patients who visited the outpatient clinic (100%) of the nephrology department of the University Hospital RWTH Aachen (Medical Clinic 2) were included in the period from July 2025 until December 2025. For the control group, peripheral blood samples were obtained from sex‐ and age‐matched volunteers who presented to the local blood bank in the city center for routine blood donation and met standard blood‐donor eligibility criteria, as described for cohort 1. These donors were anonymized upon study inclusion with only age and sex recorded. Samples were only included in the study if blood processing could be started within max. 3 h post‐sampling (in average after 1.5 h ± 48 min (SD)). The cardiovascular health status, comorbidities and medication use in this cohort were jointly assessed and summarized by the recruiting nephrologists (S.F., C.H.) and the cardiologists who supported recruitment and patient information summary of the first CKD cohort (A.V.A., B.K.), thereby minimizing reporting strategies between the two CKD cohorts.

### 5.2 CVD Patient Population

For ELISA readouts, a cohort of patients with CVD but an eGFR ≥ 60 mL/min/1.73 m^2^ was included. The patients were recruited through the cardiology department of the University Hospital RWTH Aachen from August 2023 until May 2024, as described previously [55]. The CVD phenotype included atherosclerotic CVD, heart failure, valvular heart disease with cardiovascular risk factors and cardiometabolic comorbidities present. Patients for study inclusion needed to be hemodynamically stable and ≥ 18 years of age. Furthermore, similarly as for the analyzed CKD cohort, CVD patients were not eligible for study inclusion if they had a hemoglobin value < 10 g/dL, an active infection or malignancy.

### 5.3 Whole Blood FACS Analysis of Neutrophil Phenotype and Reactivity

Citrate anticoagulated blood was directly processed upon blood collection. Blood samples were diluted 1:25 with Human TruStain FcX Fc receptor blocking solution (Biolegend) and incubated for 5 min at room temperature (RT) to reduce nonspecific staining through FcRs‐mediated Ig binding. Subsequently, the diluted blood samples were left unstimulated or were activated with interleukin 8 (CXCL8, 50 ng/mL f.c.) or phorbol 12‐myristate 13‐acetate (PMA, 50 ng/mL f.c.) for 30 min at RT. Simultaneously, samples were stained with antibodies directed against CD15 (FITC), CD45 (APC‐Cy7), CD11b (PE) and CD14 (PE‐Cy7) to identify neutrophils from the total cell population. Within this antibody mix, activation markers were assessed one by one using APC‐conjugated antibodies directed against CD18 (Biolegend 1:25), CD63 (Biolegend 1:25), CD66b (ThermoFisher 1:25), CD10 (Biolegend 1:25), CXCR2 (Biolegend 1:25), and CXCR4 (Biolegend 1:25). Additionally, fluorescence minus one (FMO) control and an overall negative control were taken along per blood sample for each condition. After stimulation and staining, samples were centrifuged for 5 min at 300 g, supernatant was removed, and cells were fixed in a 4% PFA solution for 10 min at RT. Subsequently, samples were washed in Hanks' buffer and resuspended in red blood cell (RBC) lysis Buffer (Invitrogen) for 15 min to lyse erythrocytes. Samples were washed once more, and the cell pellets resuspended in 170 μL Hanks' buffer without phenol red for measurement. Flow cytometric assessment was performed with a FACS CANTO II (BD Biosciences). Per analysis, 30.000 CD45 positive events were recorded. Analysis of flow cytometry data was performed with FlowJo version 10.8.1 (BD Biosciences). Neutrophil gating was as shown in Figure S1.

For neutrophil marker analysis, the geometric mean fluorescence intensity (gMFI) of each sample in the APC channel was measured and signal intensity was normalized by subtracting the gMFI of the corresponding FMO control. For analyzing the expression of CD11b (PE), signal intensity as gMFI was normalized by subtracting the gMFI of the CD11b‐negative cell population. Overall higher APC‐MFI values in the 2nd compared to the 1st CKD cohort are due to altered FACS instrument settings after instrument maintenance and/or a different antibody lot nr between the two recruitment periods.

The count of CD10^positive^ and CD10^negative^ neutrophils was based on the gated CD10^positive^ and CD10^negative^ neutrophils (as % of all neutrophils) and the total neutrophil count (Sysmex data) of the respective control or CKD patient.

### 5.4 Neutrophil Isolation

Neutrophils were isolated from lithium‐heparin anticoagulated blood using a Polymorph Prep density gradient. Whole blood was layered in a 1:1 ratio on top of Polymorph Prep (Progen) and centrifuged at 500 *g* for 30 min without brake. The band of polymorphonuclear leukocytes was collected. Subsequently, cells were gently washed in PBS containing 1% BSA, centrifuged at 300 *g* for 5 min and resuspended in 1 mL RBC lysis Buffer (Thermofisher) for 90 s. Lysis was stopped by addition of phenol free RPMI 1640 medium with 0.1% BSA. Cells were gently washed a final time and resuspended in phenol free RPMI 1640 medium with 0.1% BSA at a count of 2 × 10^6^ cells/mL. Isolated neutrophils were immediately used for DNA release assays.

### 5.5 DNA Release

DNA release was assessed using a Sytox green staining on isolated peripheral blood neutrophils with red blood cell lysis. A total of 300 000 neutrophils was added per well of a 96‐well plate. Neutrophils were left untreated or were stimulated with CXCL8 (50 ng/mL f.c.) or PMA (50 ng/mL f.c.) in the presence of 1 μM Sytox green at 37°C and 5% CO_2_ for up to 4 h. Cells lysed with 0.5% Triton‐X served as positive control and indication of the “maximum DNA release”. Nuclei were counterstained with 5 μM Hoechst 33342. Fluorescence intensity was measured at “0 h” as well as after 3 h using a BioTek Cytation 5 Cell Imaging Multimode Reader (Agilent). Extracellular DNA release measured as fluorescence intensity was then expressed as percentage of the maximal DNA release upon Triton‐X incubation, and values of “0 h” were accordingly subtracted to calculate the amount of extracellular DNA released over the specific time range by either unstimulated (basal) or stimulated neutrophils.

### 5.6 Assessment of MPO, Neutrophil Elastase, S100A8/A9 Levels, CXCL12, CRP, and NT‐proBNP

Plasma levels of MPO, neutrophil elastase and the S100 calcium‐binding protein A8/A9 (S100A8/A9) protein complex were determined using commercially available ELISAs (MPO: #501410, Cayman) (1:40 predilution of EDTA‐plasma); neutrophil elastase: #BMS269, Invitrogen (1:50 predilution of EDTA‐plasma); S100A8/A9: #DS8900, Bio‐Techne (1:200 predilution of EDTA‐plasma); CXCL12: #DSA00, R&D systems (1:2 predilution of EDTA‐plasma). The ELISAs were performed according to the manufacturers' instructions. NT‐proBNP and high‐sensitivity C‐reactive protein (CRP) were determined at the Laboratory Diagnostics Unit of the University Hospital RWTH Aachen using the Elecsys proBNP II kit (Roche) and the Tina‐quant Cardiac high sensitivity CRP III kit and Cobas c analyzer (Roche), respectively.

### 5.7 LEGENDplex Human Inflammation Panel 1 (13‐Plex)

To simultaneously quantify human inflammatory cytokines and chemokines in plasma, a LEGENDplex human inflammation panel 1 assay was conducted using EDTA‐anticoagulated plasma. The assay was performed according to the manufacturer's instructions. EDTA‐anticoagulated plasma was prediluted 1:1 in assay buffer. Samples were measured using a FACS CANTO II (BD Biosciences). 3000 events were recorded. Samples were read as singlets, standards in duplicate. Data was evaluated using the LegendPlex software v 2024‐06‐15 (59168) (BioLegend).

### 5.8 Statistical Analysis

Normality of the data was assessed using the Shapiro–Wilk test and data were accordingly illustrated as median with interquartile range (for non‐parametric data) or mean ± SD (for parametric data). As appropriate, non‐parametric and parametric analyses were performed as reported in the figure legends. For statistical analysis including outlier identification over the ROUT outlier test and graphical visualization, Graphpad Prism v10.3.1 was used.

## Author Contributions

S.V.: methodology, investigation, data curation, formal analysis, visualization, writing – original draft, writing – review and editing; A.A.: resources (patient inclusion), formal analysis, writing – review and editing; C.V.C.J., S.H., P.M., J.Z., C.S., J.L.: investigation, formal analysis and visualization, writing – review and editing; B.K., C.H., S.F., M.W., R.K., J.F., F.K., N.M.: resources (study participant inclusion), writing – review and editing; O.S., J.J., U.S.‐S., C.G., Y.D.: writing – review and editing; C.C.F.M.J.B.: methodology, investigation, formal analysis, visualization, supervision, validation, writing – review and editing; H.N.: project administration, conceptualization, funding acquisition, methodology, supervision, validation, writing – review and editing.

## Funding

This work was financially supported by the German Research Foundation (DFG) Project‐ID 322900939 SFB/TRR219 (S‐03, C‐01, C‐02, C‐05, M‐05 and M‐07 to J.J., H.N., R.K., N.M., C.G., and F.K.) and SFB1739 Project‐ID 546544928 (to H.N., U.S.‐S., R.K., J.J., N.M.), the Interdisciplinary Centre for Clinical Research within the faculty of Medicine at the RWTH Aachen University (PTD 1‐11 to F.K. and PTD 1‐12 to H.N.) and Project‐ID 520275106 Emmy Noether Research Group (to F.K.). The study was also supported by EFSD and Boehringer Ingelheim European Research Programme on “Multi‐System Challenges in Diabetes, Obesity and Cardiometabolic Disease” 2024 (to H.N.). Further funding was provided by the “Else Kröner‐Fresenius‐Stiftung” (Project 2020_EKEA.60 to H.N.), the German Centre for Cardiovascular Research (DZHK‐B23Ex to H.N. and Y.D.), the Alexander von Humboldt Foundation (postdoctoral fellowship to C.V.C.J.) and RWTH Start‐Up funding (StUpPD_436‐23 to C.V.C.J.). F.K. was additionally funded by the European Research Area Network on Cardiovascular Diseases (ERA‐CVD and BMBF, Grant No. JTC‐2019, MyPenPath—01KL2004) and the European Foundation for the Study of Diabetes (EFSD)/Novo Nordisk Foundation (NNF2OSA0066111). O.S. is supported by the Deutsche Forschungsgemeinschaft (CRC TRR332, A2 & Z1, CRC1123 A6, CRU342 P1, SO876/16‐1), the IZKF Münster, the Leducq Foundation, and Novo Nordisk. C.C.F.M.J.B. reports funding by the Deutsche Stiftung für Herzforschung (F/61/23).

## Ethics Statement

The study was approved by the local Medical Ethics Committee (EK418/21, EK473/21, University Hospital RWTH Aachen).

## Consent

Written informed consent according to the Declaration of Helsinki was obtained from all participants. Upon consent, CKD patients who visited the outpatient clinic of the cardiology or nephrology department of the University Hospital RWTH Aachen (Medical Clinic 1 and 2) or who were hospitalized at the cardiology department, were included in the period from December 2023 until June 2024 (cohort 1) and from July 2025 until December 2025 (cohort 2).

## Conflicts of Interest

H.N., J.J., and N.M. are founding shareholders of AMICARE Development GmbH. B.K. has served as a speaker for Novo Nordisk, Lilly, Apontis Pharma, SphingoTec, 4TEEN4 Pharmaceuticals, DGK‐Akademie, and the Professional Association of German Internists (BDI e.V.), has consulted for Novo Nordisk and Roche Diagnostics, has received travel support from Novo Nordisk, Lilly, Boehringer Ingelheim, AstraZeneca, Apontis Pharma, Daiichi Sankyo, Edwards Lifesciences, Medtronic, 4TEEN4 Pharmaceuticals, the German Society of Lipidology, DGK‐Akademie and the German Cardiac Society and has received research‐related in‐kind services from SphingoTec and 4TEEN4 Pharmaceuticals. F.K. has served as a speaker for Novo Nordisk, Boehringer Ingelheim, Bayer, Amgen, Apontis Pharma, Eli Lilly, AstraZeneca, C.T.I., Cogitando, Synlab, CSL Vifor, DGK‐Akademie, SphingoTec, 4TEEN4 Pharmaceuticals and diaplan GmbH, consulted Novo Nordisk, Eli Lilly, Bayer, Apontis Pharma, Pricewaterhouse Coopers/Strategy, received travel support from Amgen, Novo Nordisk, Boehringer Ingelheim, Bayer, SphingoTec, 4TEEN4 Pharmaceuticals, diaplan GmbH, C.T.I., Cogitando, and Eli Lilly and has received research‐related in‐kind services from SphingoTec and 4TEEN4 Pharmaceuticals. O.S. consults Roche and Novo Nordisk and receives funding from Novo Nordisk. S.F. has served as speaker or consultant for AstraZeneca, Medice, Bayer, Novartis and Stadapharm. The other authors declare that they have no competing interests.

## Supporting information


**Figure S1:** Gating strategy for analyzing neutrophils in peripheral whole blood by flow cytometry. Single cells were gated over FSC‐W/FSC‐H as well as FSC‑H/FSC‑A. Neutrophils were defined as CD45+/CD11b+/CD15+ cells. Monocytes were identified as CD45+/CD11b+/CD15‐/CD14+ cells. Eosinophils were identified as CD45+/CD11b+/CD15^intermediate^, resulting in a clear separation from neutrophils in FSC‐SSC. Eosinophil numbers correlated strongly with eosinophil numbers determined by Sysmex analysis (*r*: 0.87, *p*‐value < 0.0001, using two‐tailed Pearson correlation coefficients test).
**Figure S2:** Correlation analysis of immature CD10^negative^ neutrophil count with markers in blood and patient clinical characteristics (Cohort 1 of CKD3/4 patients and controls). Correlation analysis of CD10^negative^ neutrophil count in peripheral blood with (A) neutrophil‐borne proteins (neutrophil elastase, MPO, S100A8/A9 complex) in plasma; (B) inflammatory markers (high‐sensitivity CRP, IL6) and chemokine CXCL12 in plasma; or (C) patient clinical characteristics (eGFR, months after CVD (available for patients only)) and eosinophil count. “Months after CVD event” records months after acute CVD event as either non‐fatal myocardial infarction or stroke. Two‐tailed Pearson correlation coefficients test. CVD, cardiovascular disease; eGFR, estimated glomerular filtration rate, CRP, C reactive protein; IL6, interleukin 6; MPO, myeloperoxidase; S100A8/A9, S100 calcium‐binding protein A8/A9 protein complex. Previously identified outliers for either readout using the ROUT test (as in Figure 2) were not included in the correlation analysis.
**Figure S3:** Correlation analysis of neutrophil count with markers in blood and patient clinical characteristics (Cohort 1 of CKD3/4 patients and controls). Correlation analysis of CD10 expression (as geometric mean fluorescence intensity, MFI) on the surface of peripheral blood neutrophils with (A) neutrophil‐borne proteins (neutrophil elastase, MPO, S100A8/A9 complex) in plasma; (B) inflammatory markers (high‐sensitivity CRP, IL6) and chemokine CXCL12 in plasma; or (C) patient clinical characteristics (eGFR, months after CVD (available for patients only)) and eosinophil count. “Months after CVD event” records months after acute CVD event as either non‐fatal myocardial infarction or stroke. Two‐tailed Pearson correlation coefficients test. CVD, cardiovascular disease; eGFR, estimated glomerular filtration rate, CRP, C reactive protein; IL6, interleukin 6; MPO, myeloperoxidase; S100A8/A9, S100 calcium‐binding protein A8/A9 protein complex. Previously identified outliers for either readout using the ROUT test (as in Figure 2) were not included in the correlation analysis.
**Figure S4:** Correlation analysis of plasma markers in blood with age independent of CKD. Correlation analysis of age with plasma levels of neutrophil elastase, myeloperoxidase (MPO) and S100A8/A9 for the healthy control cohort 1 (green dots; *n* = 8–9), healthy control cohort 2 (green squares; *n* = 9), and the CVD patient cohort (blue dots; *n* = 12). The age range of the CVD cohort was comparable to that of the CKD cohorts, but CVD patients had an eGFR ≥ 60 mL/min/1.73 m^2^. Data points illustrated as dots were measured simultaneously after the first recruitment, whereas data points illustrated as squares were measured after the second recruitment. Two‐tailed Pearson correlation coefficients test. Previously identified outliers for either readout using the ROUT test (as in Figure 2) were not included in the correlation analysis.
**Figure S5:** Correlation analysis of plasma markers and CD10^negative^ neutrophils in blood with age in CKD. Correlation analysis of age with plasma levels of neutrophil elastase, myeloperoxidase (MPO), S100A8/A9 and CD10^negative^ neutrophil count for the CKD patient cohort 1 (*n* = 18–20). Two‐tailed Pearson correlation coefficients test.
**Figure S6:** Comparison of CKD patients without versus with diabetes (Cohort 1). (A) Quantification of CD10 and CXCR4 on the surface of neutrophils in blood by flow cytometry, either in unstimulated conditions or upon stimulation with CXCL8 (50 ng/mL, 30 min) or phorbol 12‐myristate 13‐acetate (PMA, 50 ng/mL, 30 min). Displayed as mean ± SD of the geometric mean fluorescence intensity (gMFI) after subtraction of the respective fluorescence minus one (FMO) control. *n* = 11 for CKD3/4 without diabetes, *n* = 10 for CKD3/4 with diabetes. (B) Quantification of neutrophil‐borne neutrophil elastase, myeloperoxidase (MPO) and the S100A8/A9 complex in plasma by ELISA, displayed as mean ± SD. (C) High‐sensitivity C‐reactive protein (CRP) in plasma, displayed as mean ± SD. (D) Interleukin 6 (IL6) in plasma by ELISA, displayed as median with interquartile range. (A–C) Parametric data; (A) Mixed‐effect analysis with Tukey's multiple comparisons test, as appropriate; (B, C) Parametric data: Welch's *t*‐test. (D) Non‐parametric data: Mann–Whitney *U*‐test. ns, not statistically significant.
**Figure S7:** Analysis of isolated neutrophils and neutrophil DNA release in patients with moderate to advanced CKD (Cohort 1). (A, B) Gating strategy for analyzing the purity of isolated neutrophils from peripheral blood by flow cytometry. Single cells were gated over FSC‐W/FSC‐H as well as FSC‐H/FSC‐A. Neutrophils were defined as CD45+/CD15+/CD16+ cells. Eosinophils were defined as CD45+/CD11b+/CD15^intermediate^. Monocytes were gated as CD45+/CD11b+/CD15‐/CD14+. Lymphocytes were defined as CD45+/CD11b‐ cells. Representative display of cell identity within the CD45+ cells as pie chart, with indication of cell type in % (representative for *n* = 3). (C) Quantification of DNA release by isolated peripheral blood neutrophils using the Sytox assay, either in unstimulated conditions or upon stimulation with PMA (50 ng/mL) or CXCL8 (50 ng/mL) for 3 h. Displayed is mean ± SD. Parametric data: Mixed‐effect analysis with Tukey's multiple comparisons test as appropriate, *n* = 6 for Healthy, *n* = 9–10 for CKD3/4. Comparison of stimulated to unstimulated cells within each patient/control group: indicated by *. ***p < 0.001; ****p < 0.0001. (D) Sytox staining of neutrophils directly after isolation and red blood cell lysis, either for healthy controls or CKD patients. As positive control, cells were lysed with 0.5% Triton‐X. Displayed is the fluorescence intensity of Sytox green normalized to the background, as well as a representative image of neutrophils (stained with Sytox (green) and Hoechst (blue)) from a healthy control donor, untreated (left) and CKD donor, untreated (right). Scale bar = 100 μm. Parametric data, statistical analysis using Brown‐Forsythe ANOVA test with Dunnett's multiple comparison, as appropriate. ****p < 0.0001. *n* = 6 for healthy, *n* = 10 for CKD3/4, *n* = 16 for positive control.

## Data Availability

The data that support the findings of this study are available on request from the corresponding author. The data are not publicly available due to privacy and ethical restrictions.
