# Supplementary material for: Immature Neutrophil Phenotype and Enhanced Myeloperoxidase in Male Patients With Chronic Kidney Disease and Cardiovascular Comorbidity
Source: Acta Physiol (Oxf). 2026 Aug 18;242(9):e70290. doi: 10.1111/apha.70290 (PMC13485703; doi:10.1111/apha.70290)
Supplement: Supplementary file 1 [file APHA-242-e70290-s001.docx]

**SUPPLEMENTAL INFORMATION TO:**

**Immature neutrophil phenotype and enhanced myeloperoxidase in male patients with chronic kidney disease and cardiovascular comorbidity**

Vondenhoff et al.

7 supplementary figures

**Supplementary figures**

**Supplemental Figure 1.** **Gating strategy for analyzing neutrophils in peripheral whole blood by flow cytometry.** Single cells were gated over FSC-W/FSC-H as well as FSC‑H/FSC‑A. Neutrophils were defined as CD45+/CD11b+/CD15+ cells. Monocytes were identified as CD45+/CD11b+/CD15-/CD14+ cells. Eosinophils were identified as CD45+/CD11b+/CD15^intermediate^, resulting in a clear separation from neutrophils in FSC-SSC. Eosinophil numbers correlated strongly with eosinophil numbers determined by Sysmex analysis (r: 0.87, *P-value* < 0.0001, using two-tailed Pearson correlation coefficients test).

**Supplemental Figure 2. Correlation analysis of immature CD10^negative^ neutrophil count with markers in blood and patient clinical characteristics (Cohort 1 of CKD3/4 patients and controls).** Correlation analysis of CD10^negative^ neutrophil count in peripheral blood with **A)** neutrophil-borne proteins (neutrophil elastase, MPO, S100A8/A9 complex) in plasma; **B)** inflammatory markers (high-sensitivity CRP, IL6) and chemokine CXCL12 in plasma; or **C)** patient clinical characteristics (eGFR, months after CVD (available for patients only)) and eosinophil count. ‘Months after CVD event’ records months after acute CVD event as either non-fatal myocardial infarction or stroke. Two-tailed Pearson correlation coefficients test. *CVD, cardiovascular disease; eGFR, estimated glomerular filtration rate, CRP, C reactive protein; IL6, interleukin 6; MPO, myeloperoxidase; S100A8/A9, S100 calcium-binding protein A8/A9 protein complex. Previously identified outliers for either readout using the ROUT test (as in Figure 2) were not included in the correlation analysis.*

**Supplemental Figure 3. Correlation analysis of neutrophil count with markers in blood and patient clinical characteristics (Cohort 1 of CKD3/4 patients and controls).** Correlation analysis of CD10 expression (as geometric mean fluorescence intensity, MFI) on the surface of peripheral blood neutrophils with **A)** neutrophil-borne proteins (neutrophil elastase, MPO, S100A8/A9 complex) in plasma; **B)** inflammatory markers (high-sensitivity CRP, IL6) and chemokine CXCL12 in plasma; or **C)** patient clinical characteristics (eGFR, months after CVD (available for patients only)) and eosinophil count. ‘Months after CVD event’ records months after acute CVD event as either non-fatal myocardial infarction or stroke. Two-tailed Pearson correlation coefficients test. *CVD, cardiovascular disease; eGFR, estimated glomerular filtration rate, CRP, C reactive protein; IL6, interleukin 6; MPO, myeloperoxidase; S100A8/A9, S100 calcium-binding protein A8/A9 protein complex. Previously identified outliers for either readout using the ROUT test (as in Figure 2) were not included in the correlation analysis.*

**Supplemental Figure 4.** **Correlation analysis of plasma markers in blood with age independent of CKD.** Correlation analysis of age with plasma levels of neutrophil elastase, myeloperoxidase (MPO) and S100A8/A9 for the healthy control cohort 1 (green dots; n=8-9), healthy control cohort 2 (green squares; n=9), and the CVD patient cohort (blue dots; n=12). The age range of the CVD cohort was comparable to that of the CKD cohorts, but CVD patients had an eGFR≥60 ml/min/1.73m^2^. *Data points illustrated as dots were measured simultaneously after the first recruitment, whereas data points illustrated as squares were measured after the second recruitment. Two-tailed Pearson correlation coefficients test. Previously identified outliers for either readout using the ROUT test (as in Figure 2) were not included in the correlation analysis.*

**Supplemental Figure 5. Correlation analysis of plasma markers and CD10^negative^ neutrophils in blood with age in CKD.** Correlation analysis of age with plasma levels of neutrophil elastase, myeloperoxidase (MPO), S100A8/A9 and CD10^negative^ neutrophil count for the CKD patient cohort 1 (n=18-20). Two-tailed Pearson correlation coefficients test.

**Supplemental Figure 6. Comparison of CKD patients without vs. with diabetes (Cohort 1). A)** Quantification of CD10 and CXCR4 on the surface of neutrophils in blood by flow cytometry, either in unstimulated conditions or upon stimulation with CXCL8 (50 ng/mL, 30 min) or phorbol 12-myristate 13-acetate (PMA, 50 ng/mL, 30 min). Displayed as mean ± SD of the geometric mean fluorescence intensity (gMFI) after subtraction of the respective fluorescence minus one (FMO) control. n=11 for CKD3/4 without diabetes, n=10 for CKD3/4 with diabetes. **B)** Quantification of neutrophil-borne neutrophil elastase, myeloperoxidase (MPO) and the S100A8/A9 complex in plasma by ELISA, displayed as mean ± SD. **C)** High-sensitivity C-reactive protein (CRP) in plasma, displayed as mean ± SD. **D)** Interleukin 6 (IL6) in plasma by ELISA, displayed as median with interquartile range. **A-C)** Parametric data; (**A**) Mixed-effect analysis with Tukey’s multiple comparisons test, as appropriate; (**B-C**) Parametric data: Welch’s t-test. **D)** Non-parametric data: Mann-Whitney U-test. *ns, not statistically significant.*

**Supplemental Figure 7. Analysis of isolated neutrophils and neutrophil DNA release in patients with moderate to advanced CKD (Cohort 1).** **A-B)** Gating strategy for analyzing the purity of isolated neutrophils from peripheral blood by flow cytometry. Single cells were gated over FSC-W/FSC-H as well as FSC-H/FSC-A. Neutrophils were defined as CD45+/CD15+/CD16+ cells. Eosinophils were defined as CD45+/CD11b+/CD15^intermediate^. Monocytes were gated as CD45+/CD11b+/CD15-/CD14+. Lymphocytes were defined as CD45+/CD11b- cells. Representative display of cell identity within the CD45+ cells as pie chart, with indication of cell type in % (representative for n=3). **C)** Quantification of DNA release by isolated peripheral blood neutrophils using the Sytox assay, either in unstimulated conditions or upon stimulation with PMA (50 ng/mL) or CXCL8 (50 ng/mL) for 3 h. Displayed is mean ± SD. Parametric data: Mixed-effect analysis with Tukey’s multiple comparisons test as appropriate, n=6 for Healthy, n=9-10 for CKD3/4. Comparison of stimulated to unstimulated cells within each patient/control group: indicated by *. ^***^*P*<0.001; ^****^*P*<0.0001. **D)** Sytox staining of neutrophils directly after isolation and red blood cell lysis, either for healthy controls or CKD patients. As positive control, cells were lysed with 0.5% Triton-X. Displayed is the fluorescence intensity of Sytox green normalized to the background, as well as a representative image of neutrophils (stained with Sytox (green) and Hoechst (blue)) from a healthy control donor, untreated (left) and CKD donor, untreated (right). Scale bar = 100 µm. Parametric data, statistical analysis using Brown-Forsythe ANOVA test with Dunnett’s multiple comparison, as appropriate. *****P*<0.0001. n=6 for healthy, n=10 for CKD3/4, n=16 for positive control.
